# Precision medicine in Parkinson’s disease patients with *LRRK2* and *GBA* risk variants – Let’s get even more personal

**DOI:** 10.1186/s40035-020-00218-x

**Published:** 2020-10-16

**Authors:** Christian U. von Linstow, Ziv Gan-Or, Patrik Brundin

**Affiliations:** 1 Center for Neurodegenerative Science, Van Andel Institute, Grand Rapids, MI 49503 USA; 2 Montreal Neurological Institute, McGill University, Montréal, QC H3A 2B4 Canada; 3 Department of Human Genetics, McGill University, Montréal, QC H3A 0C7 Canada; 4 Department of Neurology and Neurosurgery, McGill University, Montréal, QC H3A 2B4 Canada

**Keywords:** Parkinson’s disease, Precision medicine, Personalized medicine, Glucocerebrosidase, GCase, Leucine-rich repeat kinase-2, Dopamine, PD drug trials, PD risk variants

## Abstract

Parkinson’s disease (PD) is characterized by motor deficits and a wide variety of non-motor symptoms. The age of onset, rate of disease progression and the precise profile of motor and non-motor symptoms display considerable individual variation. Neuropathologically, the loss of substantia nigra dopaminergic neurons is a key feature of PD. The vast majority of PD patients exhibit alpha-synuclein aggregates in several brain regions, but there is also great variability in the neuropathology between individuals. While the dopamine replacement therapies can reduce motor symptoms, current therapies do not modify the disease progression. Numerous clinical trials using a wide variety of approaches have failed to achieve disease modification. It has been suggested that the heterogeneity of PD is a major contributing factor to the failure of disease modification trials, and that it is unlikely that a single treatment will be effective in all patients. Precision medicine, using drugs designed to target the pathophysiology in a manner that is specific to each individual with PD, has been suggested as a way forward. PD patients can be stratified according to whether they carry one of the risk variants associated with elevated PD risk. In this review we assess current clinical trials targeting two enzymes, leucine-rich repeat kinase 2 (LRRK2) and glucocerebrosidase (GBA), which are encoded by two most common PD risk genes. Because the details of the pathogenic processes coupled to the different *LRRK2* and *GBA* risk variants are not fully understood, we ask if these precision medicine-based intervention strategies will prove “precise” or “personalized” enough to modify the disease process in PD patients. We also consider at what phases of the disease that such strategies might be effective, in light of the genes being primarily associated with the risk of developing disease in the first place, and less clearly linked to the rate of disease progression. Finally, we critically evaluate the notion that therapies targeting LRRK2 and GBA might be relevant to a wider segment of PD patients, beyond those that actually carry risk variants of these genes.

## Background

Parkinson’s disease (PD) is a progressive neurodegenerative disorder, potentially with several triggers and etiologies for the pathogenic processes that converge on the accumulation of misfolded α-synuclein (α-syn) in Lewy bodies and neurites [1] and the degeneration of dopamine (DA) neurons in the substantia nigra. These processes lead to the reduced striatal DA levels and debilitating motor disturbances as a consequence [2]. In addition to the classic motor symptoms, non-motor symptoms such as rapid-eye-movement sleep behavior disorder (RBD), hyposmia, pain, constipation, orthostatic hypotension and cognitive changes are common. Some of the non-motor symptoms may precede diagnosis by several years or even decades [3]. The annual economic expenditures associated with 630,000 PD patients in the US in 2010 were estimated to be around $14.4 billion [4], and this expense is rapidly increasing given an anticipated prevalence to reach 1,238,000 cases in 2030 [5]. Therefore, developing disease-modifying treatments is of the utmost importance at present.

Symptomatic treatment of PD with, e.g., drugs targeting the dopamine system, has become increasingly “personalized” with multiple drugs and delivery systems being used according to the specific individual needs of each patient. However, when trying to achieve disease modification, a “precise” approach based on the molecular underpinnings of the disease in each patient, has not yet been fully tested. In 1–2% of PD cases, the cause of PD is attributed to the highly penetrant, autosomal dominant and recessive genes; in 5–10% of PD cases, PD is associated with strong risk genes (e.g. *LRRK2* and *GBA* mutations); and the remaining cases are idiopathic without a single identifiable cause [6]. The risk of developing PD may also depend on the initial number of DA neurons that an individual was born with [7], the combined effect of risk genes [8, 9] and environmental factors (e.g. toxins, infections, and lifestyle diseases) [10], and the advancing age that constitutes the most significant risk factor [11]. The overall heritability of PD has been estimated at around 26–36% [12], indicating the importance of environmental factors and aging. Clinical features that occur during the prodromal phase of PD, before the onset of motor deficits, often include hyposmia, constipation and depression, which may provide clues to where the disease process starts. RBD is a condition strongly associated with PD, which is coupled to a > 80% risk of developing neurodegenerative synucleinopathy within 15 years after diagnosis of the sleep disorder and is present in 30% of those who exhibit PD symptoms [13, 14]. As the origins of PD are likely to be multifactorial, it may not be surprising that the disease widely varies in the age at diagnosis, the clinical symptom profile, the rate of progression and the neuropathological features [15]. Indeed, each PD patient is unique. While symptomatic treatment that relies on the replacement with striatal DA is initially effective for most patients, the idea that the disease progression be modified by treating PD patients according to a “one-size-fits-all” approach may be fundamentally flawed [16, 17].

Several clinical trials have failed to demonstrate effective disease-modification in PD, and as mentioned above, the same disease pathway may not be relevant for all PD patients [16, 18, 19]. In addition, depending on the precise nature of the underlying pathogenic process the effective dosage of a treatment or the most relevant disease-stage might vary between individuals [15]. One reason, of many possible reasons (inappropriate target, poor target engagement, etc.), why putative disease-modifying treatments have failed in PD so far is that they might have been initiated too late. Thus, when the disease process has reached an advanced stage, it might be impossible to arrest the pathogenic cascade. Therefore, it seems attractive to initiate treatment with a potentially disease-modifying therapy during the prodromal stage, before the onset of motor symptoms [20]. Identifying patterns of biomarker change that are unique to subgroups of individuals who will further develop specific subtypes of PD would be imperative so as to identify the prodromal PD more accurately in the future [21, 22].

A “one-size-fits-only-one-or-a-few” approach considers that the pathogenic cascades involve different molecular pathways in different PD patients and suggests that the best way forward will be the precision medicine. According to the National Research Council Precision Medicine Initiative (launched in 2016), precision medicine is “*An emerging approach for disease treatment and prevention that takes into account individual variability in genes, environment, and lifestyle for each person*” [23]. Precision medicine is preferred to the older term “personalized medicine” that may be misleading by suggesting that a treatment is designed entirely for a single person [23].

PD is a model candidate for precision medicine-based approaches. Clinical trials have been underway that target specific PD risk genes and their protein products [24]. In this review, we assess the current clinical drug trials targeting LRRK2 and GBA pathways in PD. We address some of the limitations of the selected disease-targets such as the considerable heterogeneity within PD patients with *LRRK2* and *GBA* risk variants and propose how to interpret the present and the coming clinical data. Finally, we discuss if drugs that target LRRK2 and GBA can be relevant in idiopathic PD, where there is no evidence that the proteins encoded by these genes are directly perturbed.

### PD patients with *LRRK2* mutations

LRRK2 is a large multifunctional and multidomain protein expressed particularly by immune cells (e.g. microglia and macrophages) and in tissues including kidney, lung and, to a much lower extent, brain [25]. It plays important roles in inflammation [25], DA receptor trafficking [26], synaptic vesicle endocytosis [27] and protein degradation among others [28]. Several variants in the *LRRK2* gene have been associated with increased or decreased risk of PD, the autoimmune disorder Crohn’s disease, and the exacerbated immune response in leprosy [29, 30]. The most common G2019S variant accounts for up to 1% of sporadic and 4% of familial PD [31–33] and among Ashkenazi Jews as much as 10 and 28% respectively and in North African Arabs 36 and 39% respectively [34]. Other PD-associated *LRRK2* variants include R1441G/C/H, Y1699C/G [35, 36], R1628P [37, 38], G2385R [39] and I2020T [40]. Some of these variants show varied penetrance depending on the ethnicity and where the individuals live, underlining that the genetic and environmental disease-modifiers remain to be identified. Current reports of the pathophysiological mechanism behind LRRK2-PD suggest a toxic gain-of-function mechanism generated from the increased kinase activity caused by variants in the MAPKKK domain (G2019S, I2020T) or indirectly by variants in the COR domain (Y1699C/G) or ROC domain (R1441G/C/H) that reduce the GTPase activity. The LRRK2 levels in the CSF are more increased in PD patients with the G2019S risk variant [41]. The rationale behind current drug trials aiming for LRRK2 inhibition in PD is principally based on this idea [42, 43] and also on a study reporting increased wild-type LRRK2 kinase activity in idiopathic PD [44]. It has been suggested that it is desirable to reduce elevated LRRK2 in neurons in PD, but the levels of LRRK2 expression are higher in immune cells in the brain and in peripheral organs [25]. This may indicate multiple prime disease mechanisms, of which one may be more important than others. Furthermore, the multiple roles of LRRK2 and our limited understanding of the contribution of each protein domain in relation to this, may also be a simplification.

### Drug trials targeting LRRK2 hyperactivity in PD

Denali Therapeutics has recently finished a double-blinded, placebo-controlled phase Ib drug trial on a small molecule, LRRK2 inhibitor DNL201, and reported a > 50% inhibition of phosphorylated (p) LRRK2 (pS935) in blood, which is a direct measure of activity, and pRAB10, which is a downstream target of LRRK2 in peripheral blood mononuclear cells in idiopathic PD patients. The researchers also observed a 20–60% reduction in lysosomal biomarker bis-monoacylglycerol-phosphate (BMP) in urine (ClinicalTrials.gov ID: NCT03710707). This has been followed by a similar trial of the small molecule LRRK2 inhibitor DNL151 currently in phase Ib, which has shown a generally safe adverse-effect profile but also a substantial inhibitory effect on pS935 LRRK2 and pRAB10 alongside reductions in urine BMP. This study is expected to complete in Mid-2020 (ClinicalTrials.gov ID: NCT04056689). Denali Therapeutics intends to select either DNL201 or DNL151 to advance into phase 2. Ionis Pharmaceuticals is currently testing the LRRK2 antisense oligonucleotide drug BIIB094 administered intrathecally in a placebo-controlled phase I drug trial to evaluate the safety profile (ClinicalTrials.gov ID: NCT03976349). These drug trials are investigating the effects of LRRK2 inhibition in PD patients with or without *LRRK2* risk variants though challenged by the relatively low frequency of risk-variant-carriers and the even more challenging effort of recruiting patients with identical risk variants. Finally, it is interesting to note that none of the drug trials to our knowledge have considered employing non-risk-variant-carriers with base levels of LRRK2 as an important inclusion criterium though this would further refine the strategy of precision.

### Viewpoint - is LRRK2 inhibition in Parkinson’s patients sufficient?

Though fairly similar in clinical manifestation and age-at-onset of motor symptoms, PD patients with *LRRK2* risk variants seem to show, on average, milder motor and non-motor symptoms compared with idiopathic PD patients [34]. Nonetheless, the incomplete penetrance (e.g. G2019S PD: 28–74% at 59–79 years [34]) alongside the considerable variation in neuropathology within carriers of the same *LRRK2* risk variants [18, 45–47] emphasizes that additional unknown factors shape the disease phenotype. For example, in patients with *LRRK2* variants the clinical manifestations of PD may occur in the absence of Lewy bodies or other α-syn pathology, which is otherwise a disease-defining hallmark [47]. Some patients show the presence of tau-positive neurofibrillary tangles and/or senile plaques [46]. Such heterogeneity may reflect multiple disease pathways involved to varying degrees even in *LRRK2* variant-carriers. Further subclassifications of the disease in *LRRK2* variant-carriers may be warranted to develop more precise treatment in the future. A recently proposed conceptual model has suggested that LRRK2 may facilitate the development of PD and act in concert with a different trigger that actually initiates the PD process [15]. This view is in line with the reports that some asymptomatic *LRRK2* variant-carriers exhibit or develop abnormalities in the DA system including abnormal DAT and ^11^C-DTBZ (VMAT2) binding by PET imaging [48, 49]. This may indicate that the pathological changes rendering these individuals more sensitive to triggers target the DA system. Similarly, the clinical and neuropathological findings in PD patients with *LRRK2* variants are not affected by the gene-dosage [50] as observed in other PD forms (e.g. *SNCA*). Therefore, we consider that primarily *LRRK2* variant-carriers exposed to an initial trigger would develop PD. Such triggers have been proposed to include gastrointestinal microbiota perturbations, environmental toxins and pathogenic infections [15]. If such a connection exists, we contemplate that inhibiting the LRRK2 kinase activity in diagnosed PD patients may have minimal effects since the primary disease-target would have been the initial triggering event. Mechanistically, this could ensue when brain resident microglia respond to an immune trigger by engaging the LRRK2 pathways (via WAVE2) to accommodate a proinflammatory response [51]. The nigrostriatal DA neurons have exceptionally long axons, requiring a high level of energy expenditure, and have therefore been suggested to be particularly vulnerable to challenges affecting mitochondrial function [52, 53]. Given the particular sensitive phenotype of nigrostriatal DA neurons, they might be vulnerable to the release of reactive oxygen species and would be among the first cell populations to be affected. This DA neuron loss may sometimes be paralleled by aggregation of α-syn and may persist even after the infection has ceased [54]. On the other hand, if the disease process is further defined by multiple sequential hits, inhibiting LRRK2 during these events may prove most effective in protecting the already stressed DA neurons. Certain degree of microglial priming is likely to occur, which further aggravates the disease and sensitizes the host to later infections [55]. Though elevated LRRK2 activity in PD is suggested to be involved in exacerbated immune response, other functions such as the lysosomal stress response, synaptic vesicle recycling in DA neurons and changes in trophic support of DA neurons may also be impacted [56]. It is also likely that other pathological mechanisms may exist in addition to the increased kinase activity [57]. The LRRK2 inhibitors (DNL201 and DNL151) developed by Denali Therapeutics seem to be designed specifically with the aim of restoring the LRRK-mediated lysosomal dysfunction in PD as stated in a Press release (https://www.globenewswire.com/news-release/2020/01/14/1970308/0/en/...sitive-Results-From-Its-LRRK2-Program-for-Parkinson-s-Disease.html), at the Denali-Therapeutics website (https://www.denalitherapeutics.com/pipeline), and the Denali Therapeutics’ January 2020 report (https://denalitherapeutics.gcs-web.com/node/7361/pdf). However, it has not been stated whether Denali Therapeutics has considered the possible implications of the immune system that expresses the highest levels of LRRK2. Precisely how each of the *LRRK2* risk variants affects the many functions of LRRK2 and how kinase inhibition may differently affect these will need to be addressed in future studies.

In light of the potential importance of LRRK2 activity in immune cell function, safety profiling should also consider to what extent will patients receiving LRRK2 inhibitors be affected by infection. For instance, complete genetic inactivation of LRRK2 in vivo shows that the attenuation of a central infection may be at the expense of the efficiency of the peripheral immune system against a systemic infection [25]. Although the clinical relevance of this is uncertain, finding the optimal dosage-response and route of administration is critical. If the pathophysiological mechanism consists of an intensified immune response that produces a neurotoxic microenvironment in PD, it may very well require lifelong treatment to neutralize such aggravated immune response. Identifying asymptomatic *LRRK2* variant-carriers with a positive history of PD risk factors should be considered for prophylactic treatment with LRRK2 inhibitory drugs. Diabetes mellitus is a risk factor of PD [58–60] and drug trials using glucagon-like peptide-1 receptor agonists such as Exenatide have shown beneficial effects on off-medication motor scores [61, 62]. These observations have only become more relevant given a recent study demonstrating LRRK2’s role in insulin signaling (GLUT4 expression via RAB10) in iPSCs with the G2019S variant [63]. An interesting study found that the *LRRK2* risk variant-carriers resistant to PD had higher plasma urate levels than those with a PD diagnosis [64] and such measurement as a part of metabolic profiling approach [65] has proven important for distinguishing *LRRK2* variant-carriers at a higher risk of developing PD. A general shift in LRRK2 pathways may be an important component in disease that should be further characterized.

### PD patients with *GBA* mutations

The *GBA* gene codes for the enzyme glucocerebrosidase (GCase) that facilitates the lysosomal breakdown of sphingolipids (e.g. glucosylceramide into glucose and ceramide), and is expressed in most cells, notably in the macrophage lineage [66]. The characteristic swollen macrophages (i.e. Gaucher cells) contain accumulation of intracellular glucosylceramide and infiltrate organs, causing organomegaly in Gaucher disease [67, 68]. Further evidence has suggested extensive involvement of the adaptive immune system including B- and T-cell recruitment and maturation, respectively [69, 70]. More than 300 *GBA* variants have been associated with Gaucher disease [71] with varied degrees of nervous system involvement [72], while 130 *GBA* variants have been estimated to be linked with the PD risk [73], diversely affecting the disease risk, onset and progression depending on the mutation severity [74–76]. Some variants can also affect the risk of Lewy body dementia [77]. Depending on the population, about 5–20% of idiopathic PD cases are associated with *GBA* variants. Among Ashkenazi Jews, as many as 18–20% of PD patient have *GBA* variants associated with the elevated PD risk [78, 79]. The PD-associated variants in the *GBA* gene have been proposed to be associated with reduced GCase activity. Different *GBA* risk variants may decrease the GCase activity by different ways, including directly causing a loss of enzyme activity, failing to comply with endoplasmic reticulum (ER) quality control causing proteasomal degradation, perturbating trafficking to the lysosome due to ER or Golgi retention or the inability to properly connect with the lysosomal transporter LIMP2 or lysosomal activator protein Saposin C [80]. The rationale behind the clinical trials in PD targeting *GBA* risk variants is to correct cellular GCase deficiency. It may however also be relevant for some idiopathic PD patients since reduced GCase activity has been found in several brain regions and in the CSF of these patients [81–83]. Current approaches to correct these impairments include the use of pharmaceutical chaperones, gene therapy, enzyme activators and substrate reduction therapies. Pharmaceutical treatment of Gaucher disease with enzyme replacement therapy or GCase enhancers has proven effective, however, when repurposing these drugs for PD treatment, a major challenge comes with respect to their poor ability to cross the blood-brain barrier (BBB). This means that these drugs would be used in very high dosages compared to the treatment of Gaucher disease to ensure that sufficient drugs cross the BBB, which will raise an important objective of profiling adverse effect.

### Drug trials targeting GBA impairments in PD

The pharmaceutical company PRO.MED.CSA recently finished a phase II non-randomized and non-controlled clinical trial of the FDA-approved mucolytic and CGase chaperone Ambroxol (ClinicalTrials.gov ID: NCT02941822) [84]. They reported that the orally administered Ambroxol was detectable in blood and CSF in PD patients without any serious adverse effects after 186 days and that this was paralleled by a small reduction in GCase activity in the CSF caused by the inhibitory effects of Ambroxol at neutral pH. They also detected an increase in CSF α-syn and reduced tau in serum by ELISA, which were paralleled by improvement in the total MDS-UPDRS score (62.6 ± 32.2 versus 53.9 ± 30.3) and worsening in the NMSS score (49.3 ± 36.1 versus 60.8 ± 38.6) [84]. However, as the authors pointed out, the interpretation of these tests was difficult because of the lack of a placebo group [84]. Another Ambroxol drug trial designed to be double-blinded and placebo-controlled has been initiated by Weston Brain Institute, University of Western Ontario and London Health Sciences Centre and is currently in phase II, expecting a late-2020 completion (ClinicalTrials.gov ID: NCT02914366) [85]. Other current clinical trials targeting GBA include Sanofi’s glucosylceramide synthase inhibitor GZ/SAR402671 in a phase II double-blinded and placebo-controlled trial finishing in early 2023 (ClinicalTrials.gov ID: NCT02906020) and resTORbio’s TORC1 inhibitor RTB101 phase Ib/IIa trial. RTB101 is also under test in combination with rapamycin (Sirolimus) and will finish in late 2020 (ANZCTR ID: ACTRN12619000372189); interim results showed that the drugs are well tolerated and can cross the BBB. Prevail Therapeutics’ intracisternally administered GBA-coding AAV9 viral vector PR001A, is currently in a phase I/II double-blinded and sham-procedure controlled trial, which is expected to complete in 2026 (ClinicalTrials.gov ID: NCT04127578). Lysosomal Therapeutics is testing a small molecule GCase activator LTI-291 in a phase Ib safety trial (Trialregister.nl ID: NTR7299). The finished Ambroxol trial tested PD patients with or without *GBA* risk variants similar to the ongoing trials studying the effects of GZ/SAR402671 and RTB101, while the PR001A and LTI-291 drug trials are exclusively recruiting PD patients with *GBA* risk variants. In the ongoing Ambroxol trial, recruited PD patients are screened for the presence of *GBA* risk variants. Recruiting PD patients with *GBA* risk variants presents the same difficulties as recruiting PD patients with *LRRK2* risk variants, including low frequency of risk variant carriers and difficulty in collecting patients with identical risk variants. Further considering GCase levels as an inclusion criterium in non-*GBA* risk variant carriers would be relevant to refine the strategy of precision. It is also likely that some *GBA* risk variants, as we will address in the next paragraph, may require different types of drug intervention.

### Viewpoint - is GBA enhancement in PD patients sufficient?

Compared to the idiopathic PD, PD patients with *GBA* risk variants tend to have earlier onset and higher prevalence of non-motor symptoms, including RBD, cognitive impairments and dementia [86]. PD risk variants of the *GBA* gene show incomplete disease penetrance that increases with age (PD: 7.6–29.7% at 50–80 years [87]). Though relatively homogenous in terms of neuropathology [88], the clinical severity of the risk variant does show considerable effects on disease progression [74–76]. This may reflect some simplicity in the disease process since the rate of disease progression (and not the degree of neuropathology in the terminal stage) seems to be the only interchanging parameter in these patients. Because the increase in PD risk conferred by *GBA* mutations is small or modest, one can also speculate that changes in GBA require the presence of another external insult or trigger that can initiate the PD pathogenic process. The more aggressive nature of GBA-PD, when compared to idiopathic PD, is evident from the cases with particular early disease onset and the general additive effects of the number and type of mutations [89]. In combination with the stronger link between *GBA* variants and α-syn accumulation, this suggests that the *GBA* risk variants impact the disease process more potently than the *LRRK2* risk variants, despite having a smaller effect on the lifetime PD risk. We speculate that since disease progression is more rapid in GBA-PD patients and that the disease phenotype may hence be more susceptible to disease-modifying signals, it may be difficult to pharmaceutically intervene. Data extracted from the recent published Ambroxol trial [84] showed only modest increase in levels of α-syn in the CSF of PD patients with *GBA* variants (~ 8%). Therefore, the reported association of Ambroxol with CSF α-syn is mainly driven by patients without *GBA* variants (~ 14%), however the low sample size makes it difficult to reach a conclusion.

Exactly how efficient the chaperone functions of Ambroxol can correct different *GBA* risk variants is poorly understood. The *GBA* risk variants studied in the mentioned Ambroxol trial are mostly associated with reduced GCase activity and/or GCase ER retention (p.E326K, N370S, p.R463C and p.T369M/p.W393X) [90–94] however, it is worth noting that different *GBA* variants may require different pharmaceutical interventions. For instance, it would make little sense to use a GCase chaperone to treat PD patients with a *GBA* null variant or use enzyme activators in PD patients with a *GBA* variant that causes retention in the ER or Golgi. Such differences in *GBA* risk variants may ultimately require further precision in targeting the correct stage in which the pathobiology of GCase is involved. The principal mechanism of GBA-mediated disease seems to center around the reduced basal activity of lysosomal GCase. During steady-state conditions this may not lead to any discernable perturbations of lysosomal function, however, it may render neurons generally more susceptive to a wide range of stressors/triggers capable of upsetting this balance. Second, it may perturb the preparation of MHCI+II ligands in the lysosomes which is essential for immune cell communication [95]. A wider window of susceptibility may imply a wider range of disease triggers, which would further add to the more aggressive nature of PD in *GBA* risk variant carriers. Modelling GBA-PD in vitro by stressing *GBA*-deficient cells with α-syn similarly demonstrated key disease hallmarks including lysosomal dysfunction and α-syn propagation [96]. The propagation of α-syn in PD patients with *GBA* risk variants alongside the reduced efficiency of α-syn degradation may therefore be accompanied by some perturbations in the immune system.

It is known that the GCase activity is regulated by other factors than just the gene encoding the enzyme itself and recently, it has been suggested that some variants of the *TMEM175* gene that encodes a potassium pump regulating lysosomal pH may also affect the PD risk by affecting the GCase activity [97, 98]. A recent study also demonstrated that the PD onset in *GBA* variant carriers could be modified by the presence of variants in the *SNCA* and *CTSB* loci, and the latter may further exacerbate the lysosomal dysfunction by causing a deficiency in the lysosomal protease cathepsin B [99]. Identifying individuals with such disease-modifiers may become an important part of clinical trial design and treatment [9] alongside the established clinical markers such as RBD [100].

### Can we get even more personal?

**Fig. 1:**
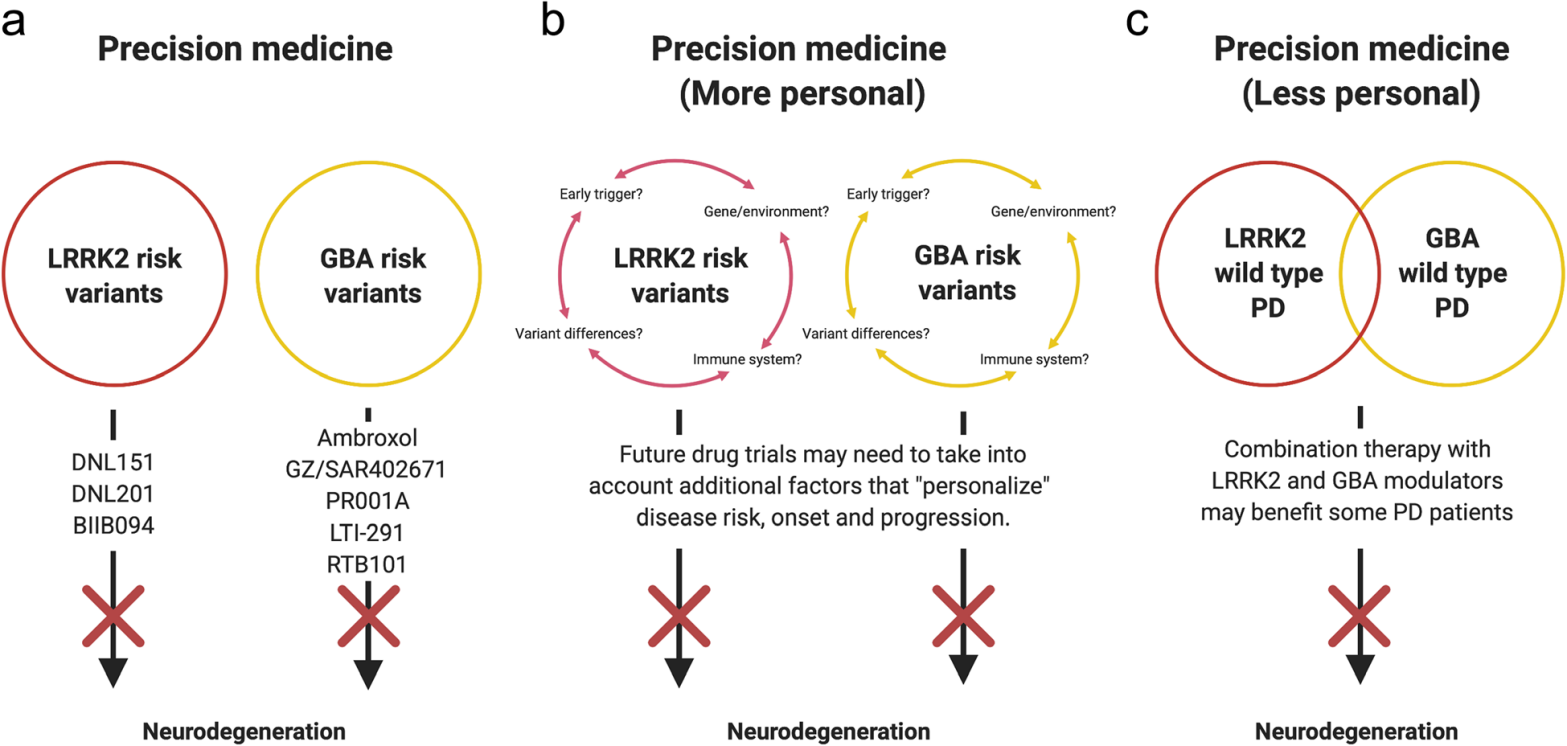
Precision medicine in current and future drug trials. **a** Current precision medicine-based therapies rely on adjusting the hyperactive LRRK2 and hypoactive GBA in PD patients with risk variants of *LRRK2* and *GBA*. **b** Several poorly understood factors including the putative disease-triggers, genes/environment, the immune system and functional differences among risk variants, may be necessary for developing more efficient and personalized therapies. **c** It is also possible that idiopathic PD patients with the same imbalances in LRRK2 and GBA as in LRRK2-PD and GBA-PD may benefit from a combinational treatment of both LRRK2 and GBA modulation

We are in the early stage of developing precision medicine-based drugs that aim to correct specific perturbations associated with single disease-associated genes (e.g. *LRRK2* and *GBA* variants). Data from drug trials generated in the next 5–10 years will resolve whether precision medicine aiming to correct the LRRK2 kinase hyperactivity and the GCase deficiency will be efficient (Fig. 1). Additional categorization of *LRRK2* and *GBA* variants may allow combinatorial or even more precise fine-tuned drug treatment (Fig. 1a). This effort may also facilitate identification of early disease triggers, as well as the understanding of the roles of the immune system (hyperactivity in LRRK2-PD and disturbances in communication in GBA-PD) and additional gene/environment disease modifiers (Fig. 1b). It will also help clarify if these drugs can be used in the treatment of a wider segment of idiopathic PD patients determined according to the levels of LRRK2 and GBA (Fig. 1c). It is likely that these drugs will at best reduce the disease progression, but not fully stop the disease process. Research on biomarkers will be crucial for early intervention, thus the biomarkers will become an essential instrument in the precision medicine “toolbox”. In addition, we propose post-hoc identification of best responders*,* which may provide guidance for further development of precision medicine.

### Can we be less personal, and get lucky?

Treating some PD patients with a combination of LRRK2 inhibitors and GCase enhancers might be a viable approach given the finding that some idiopathic PD patients exhibit LRRK2 hyperactivity together with GCase hypoactivity [44, 82, 83, 101] (Fig. 1c). If modulating LRRK2 and GCase pathways separately in LRRK2-PD and GBA-PD proves successful, one might consider that correcting both pathways could be a path forward in idiopathic PD containing these specific deficiencies, although the details of the disease pathogenesis are not well understood in those cases. However, recent observations from both clinical and in vitro studies have indicated significant differences in the disease processes among idiopathic, LRRK2- and GBA-mediated forms of PD, and that the LRRK2 and GBA pathways are differently regulated in each type. If such significant differences indeed exist, each PD type may not respond equally to LRRK2 and GCase modulatory therapies. Recently, by using metabolic brain imaging, researchers have shown that the PD patients with *LRRK2* and *GBA* variants display abnormal increases in metabolic network connectivity compared to idiopathic PD, although they have similar metabolic disease networks. Further, there are differences between LRRK2-PD and GBA-PD with regard to which network branches are the most prominently active [102]. PD patients with the *LRRK2* risk variant G2019S display increased GCase activity in their blood, which is even higher than that in healthy controls [81]. This is supported by two clinical studies on a total of 39 PD patients with both *GBA* and *LRRK2* variants, which also did not suggest a deleterious effect of *LRRK2* variants on the GCase activity [103, 104]. If *LRRK2* variants indeed lead to decreased GCase activity in patients, we would expect that these patients who have both *GBA* and *LRRK2* variants have an even more severe disease than those who carry *GBA* variants only. Surprisingly, they had a milder disease [103, 104], which supported the findings linking *LRRK2* variants to increased rather than decreased GCase activity. In primary mouse astrocytes with the *GBA* variant D490V, the resulting 90% reduction in GCase activity was paralleled by reduced LRRK2 activity. Treating cells with the LRRK2 inhibitor MLi-2 restored to some extent the lysosomal function, suggesting a compensatory upregulation of still functioning lysosomal proteins (e.g. cathepsin B) [105]. Evidence of an inverse relationship between LRRK2 and GCase activity came from a recent study showing that DA neurons derived from iPSCs procured from PD patients with the *LRRK2* risk variant G2019S had reduced GCase activity, which was reversible by treatment with the LRRK2 inhibitor MLi-2 [106]. Treatment of such iPSC-derived neurons with an GCase enhancer further increased the GCase activity [107]. Though these studies are difficult to compare, they may imply that the LRRK2 and GBA pathways are differently regulated depending on the presence of a *LRRK2* and/or a *GBA* risk variant.

## Conclusions

The axiom “*if one drug works in one type of PD it will work in all types*” has historically served well in the development of symptomatic drugs, but it has failed in the development of disease-modifying drugs in PD. We hope that the emerging field of precision medicine will help resolve this shortcoming. We believe that people with *LRRK2* and *GBA* genetic variants are eminently suited for testing new tailor-made therapies. At the same time, we recognize that there are potential limitations when targeting LRRK2 and GBA, even in PD patients who carry the genetic risk variants, most notably because we do not know in which temporal phase of the disease are the LRRK2- and GBA-related pathways important when conveying elevated PD risk. Thus, we need to go deeper (or more personal) into the disease pathogenesis of each patient when choosing therapeutic strategy. Finally, we recognize that there may be some crosstalk between the molecular cascades, although this link may not easily translate into patients due to the ageing/environment effects on lysosomal function and the immune response. Additional studies are needed to clarify the nature of LRRK2 and GBA and whether drugs targeting LRRK and GBA can potentially be combined in the more distant future.

## Data Availability

Not applicable.

## Abbreviations

α-syn: α-synuclein
BBB: Blood-brain barrier
BMP: Bis-monoacylglycerol-phosphate
*GBA*: Glucocerebrosidase
*LRRK2*: Leucine-rich repeat kinase 2
PD: Parkinson’s disease
RBD: REM sleep behavior disorder
